# Validation and cross-cultural adaptation of the Korean translation of the Achilles tendon Total Rupture Score

**DOI:** 10.1186/s12891-021-04765-w

**Published:** 2021-10-14

**Authors:** Young Hwan Park, Hyun Woo Cho, Jung Woo Choi, Hak Jun Kim

**Affiliations:** Department of Orthopaedic Surgery, Korea University Medical Center, 148 Gurodong-ro, Guro-gu, Seoul, 08308 Republic of Korea

**Keywords:** Achilles tendon rupture, ATRS, Korean, Reliability, Validity

## Abstract

**Background:**

The Achilles tendon Total Rupture Score (ATRS) is a widely used patient-reported outcome measure to assess clinical outcomes of Achilles tendon rupture, but it has not been validated in Korean yet. The purpose of this study was to translate the ATRS into Korean and evaluate its reliability and validity in a Korean population.

**Methods:**

The ATRS was translated into Korean according to recommended guidelines for forward-backward translation. Thirty-eight patients who underwent surgical treatment for Achilles tendon rupture from 2017 to 2019 were enrolled. Reliability was evaluated by the intraclass correlation coefficient (ICC), standard error of measurement (SEM), and minimal detectable change (MDC). Construct validity was assessed with Spearman rank correlations with the Korean version of the Foot and Ankle Outcome Score (FAOS) and Numeric Rating Scale (NRS) for pain in daily activity.

**Results:**

The Korean translation of the ATRS had excellent test-retest reliability (ICC = 0.84) and acceptable internal consistency (Cronbach’s alpha = 0.84). The SEM was 6.61, and the MDC was 18.32 at the individual level and 2.97 at the group level. The Korean translation of the ATRS was strongly correlated with the FASO (*r* = 0.88). Correlation with the NRS in daily activity (*r* = − 0.66) was moderate.

**Conclusion:**

The Korean translation of the ATRS showed sufficient reliability and validity for use in the Korean population.

**Level of evidence:**

II.

**Supplementary Information:**

The online version contains supplementary material available at 10.1186/s12891-021-04765-w.

## Introduction

Achilles tendon rupture is one of the most common tendon injuries in the human body. Due to greater participation in sports activities, this injury has been on the rise. Indeed, nationwide registry studies revealed that the annual incidence of Achilles tendon rupture per 100,000 people increased from 4.7 (1981) to 6.0 (1994) in Scotland, from 8.3 (1987) to 14.8 (1999) in Finland, and from 27.0 (1999) to 31.2 (2013) in Denmark [1–3]. In line with this trend, various treatments have been introduced, requiring accurate evaluation of the clinical outcomes of these treatments.

Patient-reported outcome measures (PROMs) are questionnaires that measure the status of a patient’s health condition (e.g., quality of life, symptoms, treatment effects, functioning) elicited directly from the patient, without interpretation of the patient’s response by a clinician or anyone else [4]. Because PROMs provide patient perspectives on a treatment outcome that might not be captured by clinical measurements, they play a significant role in the evaluation of prognosis and decision making for rehabilitation in Achilles tendon rupture. Among available PROMs, the Achilles tendon Total Rupture Score (ATRS) was developed to evaluate outcomes in patients treated for acute Achilles tendon rupture, and it is one of the commonly used PROMs for this condition. As a self-administered instrument, the ATRS has shown high reliability, validity, and sensitivity for measuring the outcome related to symptoms and physical activity [5]. The ATRS was originally developed in Swedish but has been validated in English, Danish, Dutch, Persian, Polish, Turkish, Greek, Norwegian, Chinese, Italian, Brazilian Portuguese, and French [6–17].

To date, no Korean PROMs have been validated specifically for Achilles tendon rupture. For the Korean population, ankle-specific PROMs such as the Foot and Ankle Outcome Score (FAOS) have been validated [18], but an Achilles tendon rupture-specific PROM is important for a more precise evaluation of treatment outcomes, especially since the incidence of this injury is growing. Therefore, the purpose of this study was to (1) translate the ATRS into Korean, (2) validate its measurement properties, and (3) compare the results from other studies. This will facilitate future research on the treatment of Achilles tendon ruptures in the Korean population.

## Methods

### Translation procedure

**Fig. 1:**
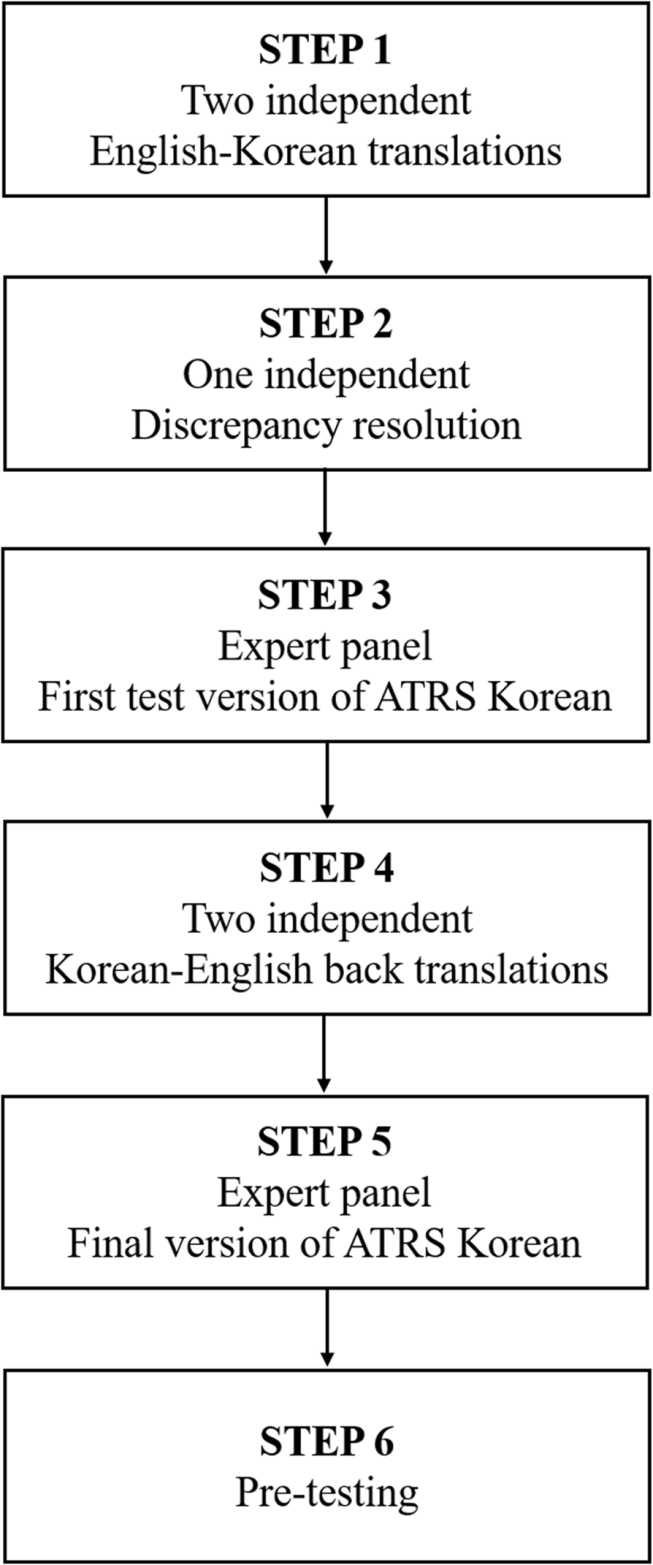
Flow chart of translation and cross-cultural adaptation of the Achilles tendon Total Rupture Score (ATRS) to Korean language

The ATRS [5] was translated into Korean according to the guidelines of cross-cultural adaptation, which standardize the translation procedures into six steps to achieve linguistic and cultural equivalence between the original and translated versions of the questionnaire [19]. Forward translations from English to Korean were performed by two independent bilingual translators, and discrepancies were resolved by judgement of a third bilingual translator. The translated ATRS was reviewed and edited by the expert panel consisting of three orthopaedic surgeons and one physiotherapist who have comprehensive experience in the treatment of Achilles tendon rupture. The backward translation into English was performed by another two independent bilingual translators. The expert panel reviewed all versions of translated ATRS and established a final version of Korean-translated ATRS (Additional file 1). A group of 15 volunteers (10 patients with Achilles tendon rupture and 5 asymptomatic adults) ensured comprehension of each question. The feedback from the volunteers revealed the questions were clear and understandable, and there were no difficulties in answering (Fig. 1).

### Study population

This study included patients who had post-discharge follow-up visits after surgical treatment for acute Achilles tendon rupture at Korea University Guro Hospital in Seoul from June 2017 to May 2019. Exclusion criteria were concomitant lower limb injury, age less than 18 years, and unable to read, write, and understand Korean. Because there is no consensus regarding sample size calculations for the validation of PROMs, we aimed to recruit as many participants as possible during the study period.

Each patient completed the questionnaires twice, with a 2-week interval, between 6 and 12 months after surgery. Because patient health status should be unchanged over the 2-week interval, patients did not receive any rehabilitation or treatment that could significantly affect their condition during the test-retest period. One of the authors evaluated the health status of patients at baseline and after 2 weeks, and patients who reported a change in their health status were excluded.

### Questionnaires

All questionnaires contained the Korean-translated version of the ATRS, a validated Korean FAOS, and Numeric Rating Scale (NRS) for pain. The ATRS questionnaire contains 10 questions, and each question is answered on an 11-point Likert scale ranging from 0 to 10. The total score ranges from 0 to 100 and is calculated by summing the individual Likert items. Higher scores indicate good recovery and lower symptoms [5].

The FAOS is a self-administered questionnaire originally designed to evaluate patients with ankle ligament injuries, but it has also been used for Achilles tendon rupture [5, 20]. The FAOS includes 42 questions with five subscales: pain, other symptoms, activities of daily living (ADL), function in sports and recreation, and foot- and ankle-related quality of life (QOL). Each question is answered on a 5-point Likert scale ranging from 0 to 4. A normalized score is calculated for each subscale, with 100 indicating no symptoms and 0 indicating severe symptoms [20].

The NRS is a commonly used assessment of pain severity. To express the intensity of pain, patients quantify their pain on an 11-point Likert scale ranging from 0 to 10, with 0 indicating no pain and 10 indicating the worst pain imaginable [21]. In this study, patients were asked to indicate the degree of pain that occurred during daily activities.

### Reliability

Test-retest reliability, which represents the stability of the scale over time, was evaluated by using the intraclass correlation coefficient (ICC) (two-way random effects model, absolute agreement definition, single measure) [22]. The ICC was judged according to the following criteria: very low (< 0.20), low (0.21–0.40), moderate (0.41–0.60), good (0.61–0.80), and excellent (0.81–1.00) [23]. The Standard error of measurement (SEM) and minimal detectable change (MDC) were calculated as follows: Standard deviation (SD) pooled = √(SD test^2^ + SD retest^2^)/2, SEM = SD pooled × √(1–ICC), MDC at the individual level = 1.96 × √2 × SEM, and MDC at the group level = (1.96 × √2 × SEM)/√n [24]. In addition, the systematic measurement error was evaluated via the Bland-Altman analysis, and mean difference and limits of agreement were calculated [25].

Internal consistency refers to the degree of homogeneity of the responses to the items of the questionnaire and was evaluated with the Cronbach alpha coefficient. A Cronbach alpha coefficient greater than 0.7 was considered to be acceptable [26].

### Construct validity

Construct validity was evaluated with correlations between the Korean translation of the ATRS and the five subscales of the FAOS and the NRS in daily activity. Correlations were measured with Spearman rank correlations and assessed with the following criteria: uncorrelated (lower than 0.4 or higher than − 0.4), moderate (between 0.4 and 0.7 or between − 0.4 and − 0.7), and strong (higher than 0.7 or lower than − 0.7) [27]. On the basis of the results of Dutch and Swedish validation studies [5, 12], we hypothesized that the Korean translation of the ATRS would be strongly correlated with the FAOS symptom, pain, function, and ADL subscales, and moderately correlated with the FAOS QOL subscale and the NRS in daily activity.

### Floor and ceiling effects

If more than 15% of responders achieve the lowest or highest possible score, floor or ceiling effects are considered to be present [27].

### Statistical analysis

Data normality was determined with the Kolmogorov-Smirnov test. Continuous variables showing a normal distribution were summarized as mean and SD. The independent t test was used to compare the continuous variables of age and BMI. The chi-square test or Fisher’s exact test was used to compare the categorical variables of sex, involved side, and activity level. Clinicometric properties were calculated as described above. Statistical analyses were performed with the Statistical Package for the Social Sciences (SPSS®) software, version 23.0 (IBM, Armonk, New York, USA) and MedCalc Statistical Software, version 20.009 (MedCalc Software bvba, Ostend, Belgium). Significance was set at *p* <  0.05.

### Ethics

This study was approved by the Ethics Committee of Korea University Medical Center (IRB No. 2019GR0477). Informed consent was obtained from all participants. All methods were carried out in accordance with Declaration of Helsinki.

## Results

### Demographics

Among 65 patients with Achilles tendon rupture during the study period, 24 were not willing to participate in the study, and three did not complete the questionnaires. Therefore, questionnaires from 38 patients were used for the statistical analysis. Patient characteristics were similar between non-participants and participants, with no significant difference observed between groups (Table 1).

**Table 1:** Patient characteristics

	Non-participants (*n* = 29)	Participants (*n* = 38)	*p*-value
Age, y	40.9 ± 11.4 (22–66)	39.2 ± 9.9 (19–56)	0.573
Sex, n (%)			0.750
Male	23 (79)	32 (84)	
Female	6 (21)	6 (16)	
BMI, kg/m^2^	22.8 ± 2.9 (18.9–25.8)	23.6 ± 3.3 (18.7–26.3)	0.684
Involved side, n (%)			0.630
Right	14 (48)	16 (42)	
Left	15 (52)	22 (58)	
Activity level			1.000
Competitive athlete	1 (3)	1 (3)	
Recreational athlete	27 (94)	35 (92)	
Nonathlete	1 (3)	2 (5)	
Time between injury and questionnaires, mo		7.6 ± 1.9 (6–12)	N/A

Data are mean ± standard deviation (range) unless otherwise noted. *BMI* body mass index, *N/A* not applicable

### Reliability

**Fig. 2:**
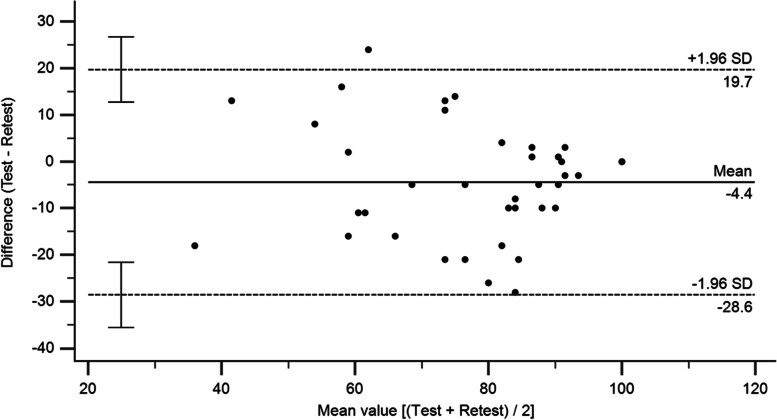
Bland-Altman plot of test–retest agreement

The ATRS was 74.8 ± 15.8 in the first test and 79.2 ± 17.1 in the retest. Two patients were excluded from the test-retest reliability analysis because they reported a change in health status between two visits. The overall ICC was 0.84 (95% confidential interval [CI]: 0.69–0.92), indicating excellent test-retest reliability. The SEM was 6.61, and the MDC was 18.32 at the individual level and 2.97 at the group level. The Bland-Altman analysis for the test-retest reliability revealed good mean agreement and narrow limits of agreement across the two visits; the mean difference was − 4.4 (95% CI: 0.37–8.47) and the limit of agreement was − 28.6 to 19.7 (Fig. 2). The Cronbach alpha coefficient was 0.84, indicating acceptable internal consistency.

### Construct validity

The overall FAOS was 64.1 ± 19.1 and details of the subscales are as follows: 64.7 ± 20.6 (pain), 67.6 ± 22.2 (other symptoms), 72.6 ± 19.7 (ADL), 43.9 ± 28.1 (function in sports and recreation), and 37.5 ± 22.8 (QOL). Table 2 summarizes the Spearman rank correlations between the Korean translation of the ATRS and the subscales of the Korean FAOS and the NRS for pain. As predicted, the Korean translation of the ATRS demonstrated strong correlations with all FAOS subscales except QOL.

**Table 2:** Construct validity measured by Spearman rank correlation coefficients

	ATRS	Correlation	*p*-value
FAOS overall	0.88	Strong	< 0.0001
FAOS symptom	0.73	Strong	< 0.0001
FAOS pain	0.81	Strong	< 0.0001
FAOS function	0.83	Strong	< 0.0001
FAOS ADL	0.79	Strong	< 0.0001
FAOS QOL	0.55	Moderate	< 0.0001
NRS in daily activity	−0.66	Moderate	< 0.0001

*ATRS* Achilles tendon Total Rupture Score, *FAOS* Foot and Ankle Outcome Score, *ADL* activities of daily living, *QOL* quality of life

### Floor and ceiling effects

None of the patients achieved the lowest score, and two patients (5.3%) achieved the highest score. Therefore, there were no floor or ceiling effects in the Korean translation of the ATRS.

## Discussion

The primary finding of this study was that the Korean translation of the ATRS showed sufficient reliability and validity. Therefore, the Korean translation of the ATRS can be used in the Korean population to evaluate the clinical outcomes of treatment for Achilles tendon rupture.

In this study, the ICC value for test-retest reliability of the Korean translation of the ATRS was 0.84, which is lower than that of previous translations of the original ATRS into other languages, including English (ICC = 0.99) [14], Persian (ICC = 0.98) [11], Turkish (ICC = 0.98) [8], Chinese (ICC = 0.98) [7], Greek (ICC = 0.97) [16], French (ICC = 0.97) [17], Italian (ICC = 0.96) [6], Brazilian Portuguese (ICC = 0.93) [10], Danish (ICC = 0.91) [13], Norwegian (ICC = 0.90) [6], Polish (ICC = 0.90) [15], and Dutch (ICC = 0.85) [12]. We suspect that the relatively low ICC value in this study was due to the timing of completion of the questionnaires, which occurred between 6 and 12 months after surgery. Majority of the patients in this study did not report changes in health status over the 2-week test-retest interval but might have experienced a marked improvement in activities compared to patients in other studies conducted later after surgery. This difference could have affected the test-retest reliability of this study. Indeed, Carmont et al. [14] evaluated test-retest reliability of the ATRS at 3, 6, and 12 months after treatment and found that reliability increased as time passed after treatment. Although the ICC value of the current study is not as high as other studies, it can still be categorized as excellent. Thus, we conclude that the Korean translation of the ATRS is reliable.

The SEM value in this study (6.63) was in agreement with that of previous studies of ATRS that documented SEM values ranging from 1.56 to 10.91 in other languages, including Brazilian Portuguese (1.56) [10], French (2.58) [17], Turkish (3.2) [8], Persian (3.57) [11], Norwegian (6.13) [6], Danish (6.67) [13], and Dutch (10.91) [12]. In addition, the %SEM value, which is an expression of the SEM as a percentage of the mean score, was 8.8% in the current study. Values of %SEM that are lower than 10% are regarded as acceptable for clinical purposes [28]. The MDC at the group and individual levels indicated that the Korean translation of the ATRS was suitable for identifying real changes when comparing groups of patients with a difference above 2.98 points and individual patients with a difference above 13.38 points.

Until now, no validated questionnaire specific to the evaluation of clinical outcomes of treatment for Achilles tendon rupture, such as the VISA-V [29] or Leppilahti score [30], has been validated in Korean. Therefore, we used the FAOS to evaluate the validity of the Korean translation of the ATRS because the FAOS has been validated in Korean and has been used to assess the outcomes of Achilles tendon rupture, as well as the validity of other translations of the ATRS [5, 6, 11, 12, 18, 31]. In previous validation studies, the correlation with the FAOS ranged from 0.6 to 0.84 in a Swedish [5], 0.72 to 0.87 in Dutch [12], 0.61 to 0.8 in Norwegian [6], and 0.55 to 0.83 in Persian populations [11]. In this study, the overall correlation between the Korean translation of the ATRS and the FAOS was above 0.7 which indicates strong correlation. Although the correlation coefficient was below 0.7 for the FAOS subscale QOL, four of five a priori hypothesized correlations were confirmed by this study. In addition, the correlation with NRS, which was assessed along with FAOS and exhibited a correlation coefficient of − 0.66, was similar with that of Dutch (− 0.58 and − 0.75, moderate to strong correlation) [12]. Therefore, considering the result of our study as comparable to those of other validation studies, we think the validity of the Korean translation of the ATRS is acceptable.

This study had three main limitations. First, the number of participants was relatively small compared to previous studies of the validity and reliability of outcome measures for ATRS. Although there is no agreed optimum method to determine the appropriate sample size for evaluating the validity of PROMs, the 38 participants in this study may be considered insufficient when compared with previous studies that enrolled a mean of 78 participants (range, 46 to 112 participants) [6–17]. Second, the responsiveness or sensitivity to change of the Korean translation of the ATRS was not assessed because no participants reported any changes in their status over the test-retest interval. Evaluating changes in patient status is critical for the assessment of therapeutic interventions, so it will be necessary confirm this in future studies of the Korean translation of the ATRS. Third, divergent construct validity was not assessed. As the FAOS was used to evaluate the Korean translation of the ATRS, we were unable to assess divergent construct validity, which has to compare with the subscales measuring different dimensions. To address this issue, additional comparisons with different dimensions such as the mental health or emotional role domain of the Short Form-36 are needed.

## Conclusion

The Korean translation of the ATRS showed sufficient reliability and validity for use in the Korean population to evaluate clinical outcomes of treatment for Achilles tendon rupture.

## Supplementary Information

**Additional file 1.** Korean translation of the Achilles tendon Total Rupture Score.

## Data Availability

The datasets used and/or analyzed during the current study are available from the corresponding author on reasonable request.

## Abbreviations

PROMs: Patient-reported outcome measures
ATRS: Achilles tendon Total Rupture Score
FAOS: Foot and Ankle Outcome Score
NRS: Numeric Rating Scale
ADL: Activities of daily living
QOL: Quality of life
ICC: Intraclass correlation coefficient
SEM: Standard error of the measurement;
MDC: Minimal detectable change
SD: Standard deviation
