# Supplementary figures and images for: Validation and cross-cultural adaptation of the Korean translation of the Achilles tendon Total Rupture Score
Source: BMC Musculoskelet Disord. 2021 Oct 14;22:876. doi: 10.1186/s12891-021-04765-w (PMC8518316; doi:10.1186/s12891-021-04765-w)

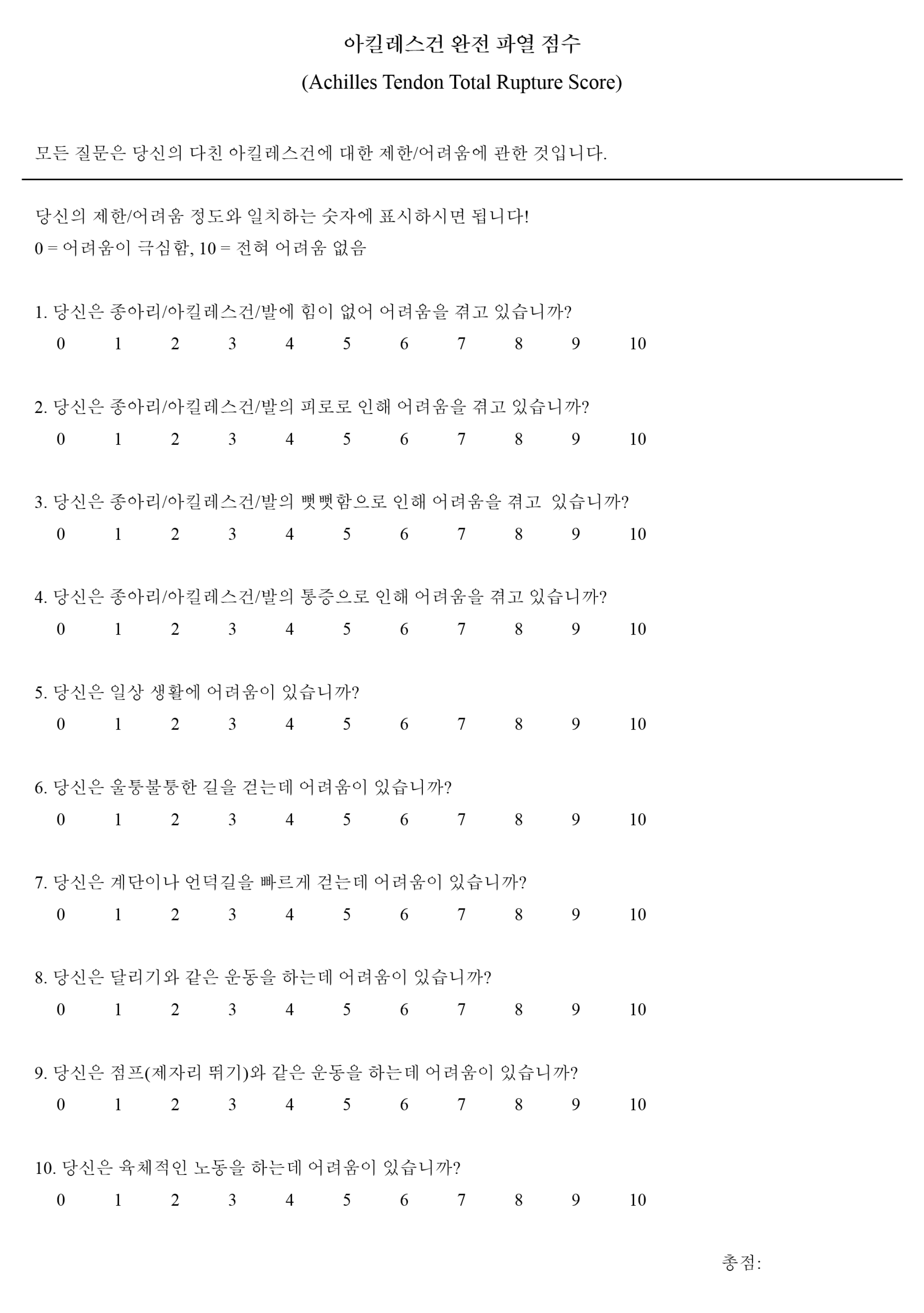

Supplement: Supplementary file 1 — Additional file 1. Korean translation of the Achilles tendon Total Rupture Score. [file 12891_2021_4765_MOESM1_ESM.zip › Appendix 1.tif]
